# Recent Trends and Potential Drivers of Non-invasive Cardiovascular Imaging Use in the United States of America and England

**DOI:** 10.3389/fcvm.2020.617771

**Published:** 2021-01-26

**Authors:** Steffen E. Petersen, Rocco Friebel, Victor Ferrari, Yuchi Han, Nay Aung, Asmaa Kenawy, Timothy S. E. Albert, Huseyin Naci

**Affiliations:** ^1^Barts Heart Centre St Bartholomew's Hospital, Barts Health National Health Service (NHS) Trust, London, United Kingdom; ^2^William Harvey Research Institute, Queen Mary University of London, London, United Kingdom; ^3^Department of Health Policy, The London School of Economics and Political Science, London, United Kingdom; ^4^Center for Global Development, London, United Kingdom; ^5^Division of Cardiovascular Medicine, Hospital of the University of Pennsylvania, Perelman School of Medicine, Philadelphia, PA, United States; ^6^Huntington Medical Foundation, Huntington Hospital, Pasadena, CA, United States

**Keywords:** imaging, nuclear cardiology and PET, diagnostic testing, computerized tomography, magnetic resonance imaging, imaging

## Abstract

**Background:** Non-invasive Cardiovascular imaging (NICI), including cardiovascular magnetic resonance (CMR) imaging provides important information to guide the management of patients with cardiovascular conditions. Current rates of NICI use and potential policy determinants in the United States of America (US) and England remain unexplored.

**Methods:** We compared NICI activity in the US (Medicare fee-for-service, 2011–2015) and England (National Health Service, 2012–2016). We reviewed recommendations related to CMR from Clinical Practice Guidelines, Appropriate Use Criteria (AUC), and Choosing Wisely. We then categorized recommendations according to whether CMR was the only recommended NICI technique (substitutable indications). Reimbursement policies in both settings were systematically collated and reviewed using publicly available information.

**Results:** The 2015 rate of NICI activity in the US was 3.1 times higher than in England (31,055 vs. 9,916 per 100,000 beneficiaries). The proportion of CMR of all NICI was small in both jurisdictions, but nuclear cardiac imaging was more frequent in the US in absolute and relative terms. American and European CPGs were similar, both in terms of number of recommendations and proportions of indications where CMR was not the only recommended NICI technique (substitutable indications). Reimbursement schemes for NICI activity differed for physicians and hospitals between the two settings.

**Conclusions:** Fee-for-service physician compensation in the US for NICI may contribute to higher NICI activity compared to England where physicians are salaried. Reimbursement arrangements for the performance of the test may contribute to the higher proportion of nuclear cardiac imaging out of the total NICI activity. Differences in CPG recommendations appear not to explain the variation in NICI activity between the US and England.

## Introduction

Many patients with suspected or known cardiovascular diseases benefit from non-invasive cardiovascular imaging (NICI) to help reach correct diagnoses, to risk stratify and to guide clinical management. Nuclear cardiac imaging and echocardiography have been long established, but more recently cardiovascular magnetic resonance (CMR) and cardiac computed tomography (CT) have become available to broaden the options available to cardiologists when considering the best investigations for their patients. All NICI techniques have advantages and disadvantages, and thus, play an important role in clinical practice. However, CMR has the benefit of combining high diagnostic accuracy with the lack of radiation, and robust diagnostic accuracy that is not affected by suboptimal image quality due to poor echo windows.

Anecdotally, England is often referred to as an exemplar of how CMR can be established as a serious contender amongst other NICI modalities. In contrast, the United States (US) is often considered to provide insufficient clinical CMR activity compared to other tests, in particular to nuclear cardiac imaging (1). Evidence to support these claims is currently lacking, recent trends in NICI use are largely unknown, and there is likely substantial geographical variation in NICI activities.

Clinical practice guidelines (CPGs) and reimbursement schemes may be two of the key determinants of trends in NICI use. CPGs have been developed for a variety of cardiovascular diseases, containing specific indications for CMR in the US (2–16) and Europe (16–28). These have been summarized by von Knobelsdorff et al. (29, 30), but since, new guidelines or updates to guidelines have been released (31–42). Between 2006 and 2011, appropriate use criteria (AUC) were developed and published—led by the American College of Cardiology Foundation (ACCF) with input from many professional bodies (43–45). Moreover, the Society for Cardiovascular Magnetic Resonance (SCMR) published five clinical situations in which CMR should not be performed as part of the Choosing Wisely program in 2014 (46). Reimbursement schemes were found to impact activity at physician-level (47, 48) and center-level (49–51).

Interestingly, financial incentives through reimbursement schemes were also found to counter CPGs recommendation, in particular if healthcare technology is substitutable. This has been highlighted in the case for cemented and uncemented hip replacements performed in the National Health Service (NHS) (52), possibly also applying to NICI technologies as, for example, a CMR scan to determine the significance of coronary artery disease could be substituted by another NICI modality, according to the CPGs.

The aim of this study was to determine trends in NICI activity for the US (fee-for-service Medicare) and England (NHS), and to assess whether variation in trends in NICI activity may be influenced by differences in CPGs and reimbursement schemes across settings.

## Methods

### Cardiovascular Magnetic Resonance and Other Non-invasive Cardiovascular Imaging Activity

Activity data for NICI in the US and England were available for people with public health insurance (Medicare fee-for-service in the US and NHS in England) for a 5-year period. Medicare data was accessed through the American College of Cardiology via a paid license, but excluded patients enrolled into Medicare Advantage Plans (53). Americans older than 65 years or living with a disability are eligible for health insurance through the federal Medicare program.

Traditional Medicare (fee-for-service) provides coverage to ~32 million Americans, while the remaining ~20 million beneficiaries are enrolled into Medicare Advantage plans (see Supplementary Material 5 for exact 2011–2015 data). Information on NICI activity was extracted and aggregated for calendar years 2011 to 2015, using relevant Current Procedural Terminology (CPT) codes (Supplementary Materials 1–4 for detail). As we cannot perform sub- analyses of NICI activities by indication, CPT codes were combined (4 for CCT, 6 for echocardiography, 4 for CMR, 11 for nuclear cardiac imaging investigations). Data are presented based on absolute numbers of activity after transformation to per 100,000 beneficiary users (Supplementary Material 5). Annual percentage change (%) in activity was calculated as the ratio of yearly activity and activity recorded in the previous year times 100.

For England, we requested extracts from the Diagnostic Imaging Dataset (DID) held by NHS Digital (54). We selected 72 SNOMED-CT codes (9 for CCT, 16 for CMR, 34 for nuclear cardiac imaging investigations, see Supplementary Material 6) from a total of 2458 SNOMED- CT codes for procedures captured in DID. Aggregate activity data was provided annually for a 5-year period, from (financial) years 2012/13 to 2016/2017, and separately for categories CMR, CCT, and nuclear cardiac imaging. Because DID does not cover echocardiography activities accurately, we used the Monthly Diagnostic Waiting Times and Activity (DM01) dataset to extract activities for echocardiography as advised by NHS Digital (55). The NHS provides healthcare services to a total of 54.3 million people and is free at the point of access. To ensure comparability with the population covered by Medicare, we restricted our denominator to 10.1 million people aged 65 years or older in England based on official statistics published by the Office for National Statistics (56). To account for the proportion of activity linked to our target population, we adjusted the absolute number of activity by 60 percentage points based on the average proportion of NICI activity linked to patients aged 65 years or older. This approach was guided by evidence from the literature and was selected because of an absence of complete activity data disaggregated by age for each NICI covered in this study (57, 58). We then transformed the adjusted number of activity to per 100,000 beneficiary users, and derived annual percentage changes (%) in activity. Sensitivity analyses investigating the impact of varying adjustment points (i.e., at 50% and 70%) on activity in England compared to the US was performed.

### Clinical Practice Guidelines

Indications relevant to CMR were collated and extracted from US-based (American Heart Association—AHA or American College of Cardiology—ACC involvement) and European- based CPGs. This was based on work published by von Knobelsdorff et al. (29, 30) but updated using latest revisions of CPGs published until 08/31/2018 (31–42). The extraction contained the class of recommendation (I, IIa, IIb, and III) and the level of evidence (A–C). In addition, we collated indications and recommendations from the American College of Cardiology Foundation (ACCF) appropriate use criteria (AUC) (43). AUC for CMR—led by ACCF with input from many other professional bodies—were included in this list of indications with extraction of the recommendation and score [A = appropriate (score 7–9), U = uncertain (score 4–6), and I = inappropriate (score 1–3)]. This indication list was complemented with the five recommendations stating when not to use CMR from the Choosing Wisely programme (46). For the combined list, we separated the indications into four categories: substitutable and recommended [Y]; substitutable and not recommended [(Y)]; not substitutable and recommended [N]; and not substitutable and not recommended [(N)]. A two-center pilot study was conducted to assess the role of guideline-based recommendations on NICI use (see Box 1).

Box 1 **Substitutability in guideline-based recommendations as a potential determinant of NICI use—a two center pilot study in the US and England.**

**Objective**: To determine whether substitutability of NICI techniques in guideline-based recommendations could be a driver of geographical variation in NICI use.

**Methods:** Consecutive CMR reports were anonymized. Blinded to the categories of whether modalities were substitutable for indications, a cardiologist (AK) independently retrospectively categorized consecutive June 2018 CMR reports from one large academic center in the US (Hospitals of the University of Pennsylvania-Penn Presbyterian) and one in England (Barts Health NHS Trust) according to the indication from any of the CPGs and AUCs. Another cardiologist (SEP) then determined the frequency of CMR scans performed compliant with the recommendations and the frequency of CMR scans performed with substitutable indications. A two-sided Chi-square test was performed to compare frequencies of CMR activity with substitutable guideline-based indications between an academic hospital in the US and in England. A *p*-value of equal or <0.05 was considered statistically significant.

**Results:** At the Hospitals of the University of Pennsylvania-Penn Presbyterian, out of 129 consecutive CMR reports, 117 reports could be categorized, and all of these were indicated (i.e., CPG class I, IIa or IIb or AUC category “appropriate” or “uncertain”). Of the 117 reports, 51 reports had substitutable indications. Thus, 43.6% of scans performed and with a categorizable indication could be substituted by a different imaging test without deviation from the guidelines. At Barts Health NHS Trust, out of 100 consecutive CMR reports assessed, 93 reports could be categorized, and all of these were indicated (i.e., CPG class I, IIa or IIb or AUC category “appropriate” or “uncertain”). Of the 93 reports, 33 reports had substitutable indications. Thus, 35.5% of scans performed and with a categorizable indication could be substituted by a different imaging test without deviation from the guidelines. These findings were not statistically different between the US and England (Chi-square = 1.419, *p* = 0.234).

**Interpretation**: Our findings suggest that clinical practice between the US and England in this CMR two-academic-center pilot may not differ with regards to the frequency of CMR use for recommended indications that did not favor one NICI technique over another. Substitutability is unlikely a major driver of variation in NICI activities.

### Cardiac Imaging Reimbursement

To match NICI activity, we collated information on NICI reimbursement covered by traditional Medicare and the NHS. For Medicare and non-invasive imaging, the description has been informed by discussions with members of the Society for Cardiovascular Magnetic Resonance (SCMR) US Working Group (VAF, TSEA), which aims to support CMR activity. Work by Ferrari et al. provided an overview into the relevant aspects of the US payment system with regards to cardiovascular imaging (1). This was complemented by 2018 tariffs outlined on the Centers for Medicare & Medicaid Services website. The final 2018 cardiac imaging payment systems can be downloaded as addendum A (59). The relevant tariffs can be found according to the codes that capture the imaging modalities (2018 Medicare Physician Fee Schedule final rule) (53, 60). For the NHS, we extracted information from NHS England (i.e., the executive non-departmental body in charge of the healthcare budget and oversight of commissioning services) and NHS Improvement (i.e., the non-departmental body in charge of service provision of local healthcare providers) 2018/19 National Tariff Payment System (61), and annex A (i.e., national prices and national tariff workbook) (62).

To assess whether physician payment is significantly different between modality reporting, insights from three cardiac imaging physicians in the US and in England were sought to estimate the number of typical reports for each modality per hour. This average reporting volume multiplied by the fee-for-service provided estimates of hourly earnings and allowed investigating whether financial incentives at the physician-level could influence the choice of NICI modality.

### Statistical Analysis

Two-sided Chi-square tests were performed to compare frequencies of CMR activity with substitutable guideline-based indications between the US and Europe. A *p*-value of equal or <0.05 was considered statistically significant. The analysis was performed using a web-based social science statistics platform (63).

## Results

### CMR and NICI Activity

**Figure 1:**
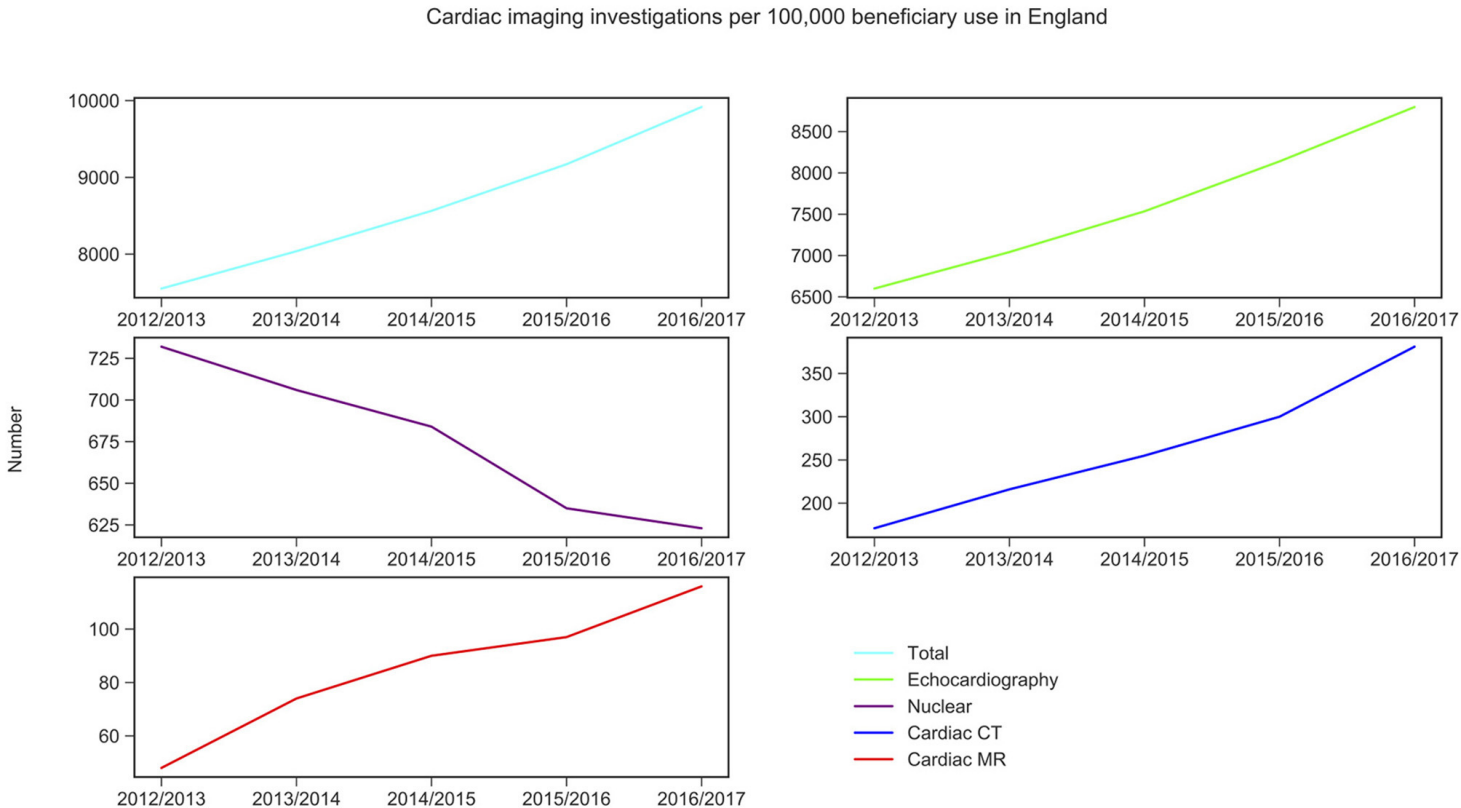
Non-invasive cardiac imaging activity in England and its change over 5 years. Source: Diagnostic Imaging Dataset, NHS Digital for Nuclear cardiac imaging, cardiac CT and cardiac MR (Snomed-CT codes used, see Supplementary Material 6); Echocardiography data was taken from the Monthly Diagnostic Waiting Times and Activity (DM01) dataset.

**Figure 2:**
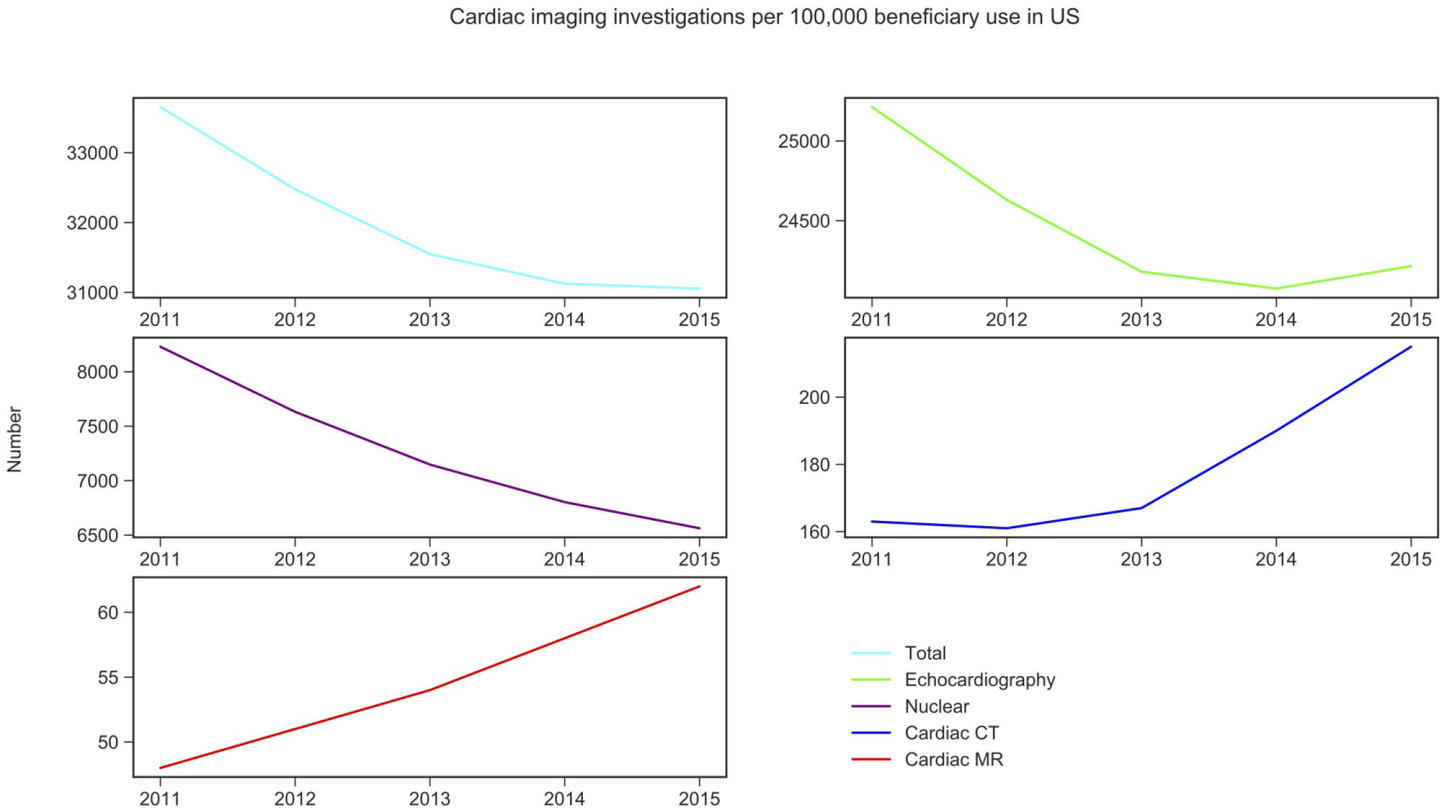
Non-invasive cardiac imaging activity in US and its change over 5 years. Source: Medicare data accessed via the American College of Cardiology. Details of which CPT codes used can be found in the Supplementary Materials 1–5.

CMR activity per 100,000 beneficiaries with public health insurance in the US (from 2011 to 2015) and for England (from 2012 to 2016) are displayed in Figures 1, 2 and Table 1. During the study period, in England, echocardiography remained the most commonly performed NICI test (87–89%), followed by nuclear cardiac imaging (6–10%), cardiac CT (2–4%), and CMR (1%).

**Table 1:** Cardiac non-invasive imaging activity per 100,000 beneficiary use for Medicare in US and NHS in England (percentage of total activity per year).

**Modality**	**USA 2011**	**England (2012/13)**	**USA 2012**	**England (2013/14)**	**USA 2013**	**England (2014/15)**	**USA 2014**	**England (2015/16)**	**USA 2015**	**England (2016/17)**
Cardiac CT	163 (0.48%)	171 (2.26%)	161 (0.50%)	216 (2.69%)	167 (0.53%)	255 (2.98%)	190 (0.61%)	300 (3.27%)	215 (0.69%)	381 (3.84%)
Cardiac MR	48 (0.14%)	48 (0.64%)	51 (0.16%)	74 (0.92%)	54 (0.17%)	90 (1.05%)	58 (0.19%)	97 (1.06%)	62 (0.20%)	116 (1.17%)
Echocardiography	25,213 (74.92%)	6,600 (87.40%)	24,631 (75.85%)	7,042 (87.60%)	24,180 (76.64%)	7,535 (87.98%)	24,074 (77.35%)	8,139 (88.74%)	24,215 (77.97%)	8,797 (88.71%)
Nuclear	8,230 (24.45%)	732 (9.70%)	7,632 (23.50%)	706 (8.79%)	7,148 (22.66%)	684 (7.98%)	6,803 (21.86%)	635 (6.93%)	6,563 (21.13%)	623 (6.28%)
Total	33,654	7,551	32,475	8,039	31,549	8,564	31,125	9,171	31,055	9,916

*The total (sum) of the components may contain rounding errors; Data sources as described in Figures 1, 2. Activity for England restricted to beneficiaries aged 65 years and older*.

Similarly, in the US, echocardiography was the most commonly performed NICI test (75–78%), followed by nuclear cardiac imaging (21–25%), cardiac CT (<1%), and CMR (<1%) (Table 1).

CMR activity is higher (0 to 1.4-fold) in England compared to the US per 100,000 beneficiaries. This is also the case for cardiac CT (1.1 to 1.4-fold). Nuclear cardiac imaging and echocardiography are two techniques more frequently performed in the US compared to England: by 10.3–10.4 times and 2.9–3.7 times, respectively (Table 1).

Trend analysis by modality and country suggested that in England the total NICI activity has increased by a factor of 1.31, with increases in all imaging modalities (by factors of 2.40 for CMR, 2.22 for cardiac CT and 1.33 echocardiography)—except nuclear cardiac imaging (reduction by 15%). Unlike in England, in the US the total NICI activity has decreased by 8%. This is driven by reductions in echocardiography (−4%) and nuclear cardiac imaging (−21%), which in absolute numbers are the largest contributors to NICI activity. Cardiac CT and CMR use have increased in the US by factors of 1.31 and 1.29, respectively, but relative growth rates are smaller compared to England.

### Clinical Practice Guidelines

The most common CMR indications are similar in the US and in Europe (i.e., including England; see Table 2). Cardiomyopathies, myocarditis, heart failure and viability are among the top three indications in the mainly US based SCMR Registry (formerly known as GCMR) (64), the EuroCMR Registry (65), and according to a 2011 BSCMR survey, among CMR centers in the United Kingdom (66).

**Table 2:** Top 3 indication categories for cardiovascular magnetic resonance in the literature.

**Indications**	GCMR (Mostly US)*	EuroCMR Registry**	BSCMR survey (England)***
TOP 1	Myocarditis/Cardiomyopathy (21%)	Coronary artery disease (34.2%)	Heart failure/Cardiomyopathy (39%)
TOP 2	Viability (16%)	Myocarditis/Cardiomyopathy (32.2%)	Viability (26%)
TOP 3	Coronary artery disease (16%)	Viability (14.6%)	Congenital heart disease (19%)

*Source*.

* *Kwong et al. (64)*.

** *Bruder et al. (65)*.

*** *Antony et al. (66)*.

The result of the extraction of individual recommendations from the European and the American CPGs and the AUC, and the statements in the Choosing Wisely program are summarized in Supplementary Material 7. The ACC/AHA have published 12 CPGs or updates between 2008 and 08/31/2018, which contain 67 specific CMR recommendations. The European Society of Cardiology (ESC) has published 15 CPGs with 68 CMR recommendations between 2010 and 08/31/2018. The AUC related to CMR contain 30 recommendations published in 2006. Amongst recommendations that were not class III, or categorized as inappropriate in AUC, or part of the five Choosing Wisely program recommendations, the following proportions were substitutable by an alternative NICI test: for ACC/AHA CPGs there were 40 out of 67 (60%), for the ESC CPGs there were 38 out of 68 (56%), and for the AUC there were 11 out of 30 (37%) recommendations. In pairwise comparisons, only the ACC/AHA recommendations had significantly more substitutable indications compared with AUC (Chi-square = 4.4, *p* = 0.036). Importantly, there was no statistically significant difference between the ACC/AHA and the ESC recommendations (Chi-square 0.202, *p* = 0.653 for ACC/AHA vs. ESC; Chi-square 3.075, *p* = 0.079 for ESC vs. AUC).

### Reimbursement Schemes for Non-invasive Cardiovascular Imaging

Medicare reimbursement schemes for NICI differ depending on the setting it is performed in, for example the hospital-based inpatient, hospital-based outpatient, or physician office/imaging center (Table 3). Inpatient investigations are reimbursed as part of hospital patient episodes according to a diagnosis-related group (DRG), thus the DRG related amount remains constant whether or not NICI was performed. In contrast, most physicians are paid separately through Medicare for the activity of image reading and reporting. Outpatient investigations in the hospital setting attract payment according to the outpatient prospective payment system (OPPS) via an ambulatory payment classification (APC). Similar to the inpatient setting, physicians involved in reading and reporting may charge Medicare separately for this professional activity (Tables 4, 5). In the third setting of performing NICI in a physician's office, or dedicated imaging facility, payment occurs in two ways: a technical component and a professional component according to the Medicare Physician Fee Schedule. There is a range of fees (i.e., represented in 2018 US Dollars) the centers attract with the largest amount for nuclear cardiac imaging (up to $1202 + physician fees). Reimbursement is lower for CMR (at $682), cardiac CT (at $253), and echocardiography (at $487), plus physician fees.

**Table 3:** Overview of non-invasive cardiac imaging reimbursement schemes from public healthcare provision.

**Setting [center/physician fees]**	**US**	**England**
Hospital inpatient [center]	DRG	HRG++
Hospital inpatient [physician]	Charges Medicare	NHS salaried
Hospital outpatient [center]	APC	National tariff
Hospital outpatient [physician]	Charges Medicare	NHS salaried
Office imaging facility [center]	MPFS technical component	N/A
Office imaging facility [physician]	MPFS professional component	N/A

*Details are listed for the US and England according to the settings and at the level of the center and the physician performing the scan and reporting*.

*DRG, diagnosis related group; HRG, healthcare resource group; MPFS, Medicare Physician Fee Schedule; APC, ambulatory payment classification*.

**Table 4:** Outpatient reimbursement for 2018.

**Setting [center/physician fees]**	**US (Hospital-based outpatient)**	**US (Office imaging facility)**	**England (NHS Hospital)**
CMR—Center	CPT 75557 and 75559 –> APC 5523: $245.22 CPT 75561 –> APC 5572: $456.34 CPT 75563 –> APC 5573: $681.83	CPT 75557: $217.88 CPT 75559: $307.08 CPT 75561: $307.08 CPT 75563: $376.19	RD08Z (non-contrast): £325 incl. £22 report RD10Z (pre- and post- contrast): £448 incl. £20 report
CMR—Physician	Charges Medicare (similar to professional component of MPFS, next column)	CPT 75557: $118.44 CPT 75559: $146.16 CPT 75561: $130.68 CPT 75563: $150.12	NHS salaried
CCT—Center	CPT 75571 –> APC 5521: $62.11 CPT 75572, 75573, and 75574 –> APC 5571: $252.72	CPT 75571: $75.60 CPT 75572: $201.96 CPT 75573: $267.84 CPT 75574: $311.40	RD28Z (Complex CT scan): £216 incl. £20 report
CCT—Physician	Charges Medicare (similar to professional component of MPFS, next column)	CPT 75571: $29.52 CPT 75572: $88.92 CPT 75573: $128.88 CPT 75574: $120.96	NHS salaried
Nuclear—Center	CPT 78452, 78453, and 78454 –> APC 5593: $1202.60	CPT 78452: $419.39 CPT 78453: $270.36 CPT 78454: $393.11	RN20Z (MPS): £159 incl. £26 report RN21Z (MPS, stress only): £216 incl. £26 report RN22Z (MUGA): £190 incl. £19 report
Nuclear—Physician	Charges Medicare (similar to professional component of MPFS, next column)	CPT 78452: $81.00 CPT 78453: $50.40 CPT 78454: $68.04	NHS salaried
Echo—Center	CPT 93350 and 93351 –> APC 5524: $486.66	CPT 93350: $139.32 CPT 93351: $151.92	RD51A-C (simple echo, age- dependent): £64–89 EY50Z (complex echo): £176 (or £430 if done as day case) EC21Z (complex echo for CHD): £179 (or £620 if done as day case)
Echo—Physician	Charges Medicare (similar to professional component of MPFS, next column)	CPT 93350: $72.72 CPT 93351: $87.12	NHS salaried

*Details are listed for the US and England according to the settings and at the level of the center and the physician performing the scan and reporting*.

*CPT, Current Procedure Technology; APC, ambulatory payment classification; CHD, congenital heart disease; CCT, cardiac computed tomography; CMR, cardiovascular magnetic resonance; MPFS, Medicare Physician Fee Schedule*.

*CMR CPT codes*.

*75557 Cardiac MR imaging for struct and morph w/o contrast*.

*75559 Cardiac MR imaging for struct and morph w/o contrast w/stress imaging*.

*75561 Cardiac MR imaging for struct and morph w/o contrast, followed by contrast, and further sequences*.

*75563 Cardiac MR imaging for struct and morph w/o contrast, followed by contrast, and further sequences, w/stress imaging*.

*Cardiac CT CPT codes*.

*75571 CT heart w/o contrast, w/quantitative eval of coronary calcium*.

*75572 CT heart w/contrast for eval of cardiac struct and morph*.

*75573 CT heart w/contrast for eval of cardiac struct and morph (congenital heart disease)*.

*75574 CT angio heart, coronary arteries and bypass grafts w/contrast*.

*Cardiac nuclear CPT codes*.

*78452 MPI, tomographic (SPECT) (inc. attenuation correction, qualitative or quantitative wall motion, ejection fraction by first pass or gated technique, additional quantification, when performed); multiple studies, at rest and/or stress (exercise or pharmacologic) and/or redistribution and/or rest reinjection*.

*78453 Myocardial perfusion imaging, planar (inc. qualitative or quantitative wall motion, ejection fraction by first pass or gated technique, additional quantification, when performed); single study, at rest or stress (exercise or pharmacologic)*.

*78454 MPI, planar (inc. qualitative or quantitative wall motion, ejection fraction by first pass or gated technique, additional quantification, when performed); multiple studies, at rest and/or stress (exercise or pharmacologic) and/or redistribution and/or rest reinjection*.

*Cardiac echo CPT codes*.

*93350 Echo rest and cv stress test w/interp and report*.

*93351 Echo rest and cv stress test w/interp and report including electrocardiographic monitoring w/physician supervision*.

**Table 5:** Estimated hourly physician Medicare reimbursement in 2018 for reporting non-invasive cardiac imaging in the US according to Medicare Physician Fee Schedule and estimated scans.

**Modality**	****~**Professional component in 2018 ($)**	Average reports/hour*	**Hourly reporting reimbursement ($)**
CMR	150	3.100	465
CCT			
	125	4.333	542
Nuclear	50	6.500	325
Echo	80	6.300	504

*Source*.

* *3 US and 3 UK non-invasive imaging cardiologists' estimate*.

*CMR, cardiovascular magnetic resonance; CCT, cardiac computed tomography; Nuclear, cardiac nuclear investigations; Echo, echocardiography*.

The NHS provides free healthcare at point of entry to ~54.3 million people in England (67). Similar to the US, reimbursement differs for NICI performed for inpatient and outpatient settings. For inpatients, the reimbursement is through payments based on the inpatient hospital episode classed according to a Healthcare Research Group (i.e., HRG++), while for outpatients, hospitals are paid the national tariff for the relevant imaging modality. National tariff 2018 prices were £448 (~$578) for CMR, £216 (~$279) for cardiac CT, £159 to £190 (~$204–$245) for nuclear cardiac imaging, and £64–£179 (~$83–$231) for echocardiography. A key difference to the US is that hospital doctors are salaried and thus not paid on a fee-for-service basis as is the case in most centers in the US (Table 4). There are no NHS imaging facilities run outside hospitals that would be reimbursed differently. Given the above prices, the relative payment at the center-level is higher for nuclear cardiac imaging and echocardiography compared to CMR and cardiac CT in the US when compared to England. This observation is more pronounced in the US hospital outpatient setting than in the office-based setting.

Although payment at the physician-level varies from salaried physicians in England to a payment system based on fee-for-service in the US, there appears to be no major driver to perform one particular NICI test over another within the US fee-for-service reimbursement schemes (Table 5). Cardiac CT attracts the highest hourly rate ($542) followed by echocardiography ($504), CMR ($465), and then nuclear cardiac scan reporting ($325). CMR hourly reporting rates were 1.4 times higher than for nuclear reporting.

## Discussion

In this study, we used Medicare fee-for-service and NHS Digital data to investigate NICI activities. We find that in 2015, the US performed over three-times more NICI compared to England (31,055 vs. 9,171 per 100,000 beneficiaries). CMR activity in the US was 0–1.5 times smaller compared to England, which contrasts with the 10.3–10.4 times higher nuclear cardiac imaging activity in the US. We find no evidence to suggest that recommendations in international CPGs may be linked to the observed differences in NICI activity. Between both countries, financial remuneration varies at the physician-level (fee-for-service vs. salaries), which may contribute to the comparatively high NICI activity in the US. Payment mechanisms for the scan component of the NICI may also be related to the relatively more frequent activity of nuclear cardiac imaging compared to the use of CMR in the US, as nuclear cardiac imaging is incentivized through higher fees (up to $1202 vs. up to $682, respectively).

CPGs do not appear to influence relative NICI use. We found American and European recommendations of CMR use to be similar in terms of total number of recommendations and proportion of recommendations in which CMR could be substituted by other NICI techniques. Contrary to our hypothesis that these substitutable indications were more frequent referral reasons in England than in the US, findings from our pilot study, focusing on data from one US- based and one England-based CMR center, show that the proportions were similar.

NICI activity was 3.4-fold higher in the US compared with England in 2015. This may partly be explained by the fee-for-service payment for physicians in the US compared to salaried doctors in England. In fact, evidence on the effect of salaries on reducing activity compared with fee-for- service payments have been found in pediatric and primary care settings (68–70). However, the payment for different NICI reports appears proportionate if calculated as an hourly rate, for example nuclear cardiac imaging reporting attracts no higher pay over CMR or cardiac CT reporting.

Although NICI activity data was available in the US (Medicare fee-for-service, excluding patients enrolled in Medicare Advantage Plan) and England (NHS), because its provision is mandatory to providers of Medicare and NHS services, this study relied on the accuracy of coding information. Population demographics are likely to vary between Medicare and NHS beneficiaries. By definition, most Medicare beneficiaries will be 65 years and older, while the NHS covers people of all ages. We addressed this concern by estimating the adjusted number of activities for NHS beneficiaries aged 65 years and older, allowing for age-wise comparisons in NICI activity between both health care systems. While an adjustment factor of 60 percent was chosen on the basis of disaggregated information on echocardiography activity from NHS Digital and CMR activity by age in England from published literature, this may not reflect the exact number of activity linked to the target population. The adjustment factor was chosen because of a lack of available activity data disaggregated by age group. Sensitivity analyses addressing variation in the adjustment factor (50 and 70%) suggested small changes to the NICI activity per 100,000 beneficiaries, but importantly, differences in activity between the US and England remained substantial (see Supplementary Material 8). Moreover, overall findings remained consistent when investigating NICI activity for the whole NHS population, rather than as a subgroup, with increases to the magnitude of difference in NICI activity between the US and England (see Supplementary Material 9). Our objective in this study was exploratory in nature; therefore, we cannot make causal inferences about the relationship between NICI activity, and potential policy predictors including financial incentives and CPGs.

Future work should address other potential determinants of NICI use and investigate whether activity numbers represent over- or under-use of these diagnostic tests. There are several determinants of NICI activity, all of which are context specific (71). Unlike echocardiography and dedicated nuclear cardiac equipment, cardiac CT and CMR activities are often competing for scanner time with non-cardiac activity (e.g., brain scans). Because this study focusses on NICI activity and reimbursement, extrapolation without this consideration to sites where magnetic resonance imaging and CT scanners are used for both cardiac and non-cardiac activity. It is possible that NICI activity is disincentivized by higher reimbursement for non-cardiac activity, particularly if the service is provided by different specialties (e.g., cardiology and radiology).

There are other important factors that influence CMR activity levels, including the lack of experienced CMR experts as well as knowledge and expectations of the referring physician.

For example, it is possible that training of reporting physicians may be lagging behind to supply sufficient certified NICI experts, in particular to perform CMR that requires substantial clinical exposure and knowledge. This presents an opportunity cost for well-versed clinicians in other NICI modalities to train in CMR. For newer indications, referring physicians may need to be trained to be referring their patients for the most appropriate NICI test. As nuclear cardiac scans are mostly performed by cardiologists in the US, self-referral for nuclear cardiac scans may be chosen to ensure the service is performed internally (e.g., due confidence in own reports, reimbursement for this service). As a consequence, the “ownership” (radiology/nuclear medicine/cardiology) of the equipment and provision of certain NICI services will determine relative NICI activity across many healthcare systems.

Differences in reimbursement schemes through fee-for-service physician payment in the US for NICI may contribute to the three-times higher NICI activity compared to England where doctors are salaried. Reimbursement arrangements for the performance of the test without reporting fees may contribute to the relatively higher proportion of nuclear cardiac imaging of the total NICI activity, which may make CMR activity seem lower in the US. We found that absolute CMR activity is higher in England than in the US, suggesting that reimbursement factors could be important drivers. Based on our analyses for a study period from 2011 to 2016, CPGs do not seem to explain the variation in NICI activity between both countries, although we cannot discount that CMR use may have increased due to an emphasis of its value in CPGs published after 2016. The presented data provides a comprehensive insight into current NICI activity, reimbursement arrangements and CPGs in the US and Europe.

## Data Availability Statement

Medicare data can be accessed under special license agreements. Hospital Episode Statistics data can be obtained via NHS Digital. The authors will be able to share access to the data used for this study upon request.

## Author Contributions

SP had the initial idea for this study. SP and RF prepared the first draft. RF made substantial contributions to the write-up and editing of the manuscript. Analysis was conducted by SP and guided by HN. Technical support and creation of figures and tables was provided by NA and AK. All authors contributed to significant edits of this manuscript.

## Conflict of Interest

SP, RF, and TA provide consultancy and have stock options for Circle Cardiovascular Imaging Inc., Calgary, Canada. RF received personal fees from AstraZeneca for consultancy work unrelated to this project. The remaining authors declare that the research was conducted in the absence of any commercial or financial relationships that could be construed as a potential conflict of interest.
