# Supplementary material for: Recent Trends and Potential Drivers of Non-invasive Cardiovascular Imaging Use in the United States of America and England
Source: Front Cardiovasc Med. 2021 Jan 26;7:617771. doi: 10.3389/fcvm.2020.617771 (PMC7870990; doi:10.3389/fcvm.2020.617771)
Supplement: Supplementary file 1 [file Data_Sheet_1.PDF]

SUPPLEMENTARY MATERIALS

S 1: Medicare cardiac MRI activity by CPT code.

CPT Code	Description	2011	2012	2013	2014	2015
75557	Cardiac MRI for morph	2,371	2,448	2,480	2,496	2,504
75559	Cardiac MRI w/ stress image	92	68	57	71	66
75561	Cardiac MRI for morph w/ dye	10,866	11,858	13,010	14,358	15,886
75563	Cardiac MRI w/ stress image & dye	2,049	2,158	2,071	2,090	2,129
	**SUM**	15,378	16,532	17,618	19,015	20,585
	**Per 100,000 Beneficiaries**	**48**	**51**	**54**	**58**	**62**

S 2: Medicare cardiac CT activity by CPT code.

CPT Code	Description	2011	2012	2013	2014	2015
75571	CT heart w/o dye w/ calcium	4,080	4,713	4,920	8,105	11,753
75572	CT heart w/ 3D image	6,280	7,918	9,842	12,368	14,695
75573	CT heart w/ 3D image, congenital	387	318	288	417	397
75574	CT angio heart w/ 3D image	41,492	39,666	39,949	41,662	44,026
	**SUM**	52,239	52,615	54,999	62,552	70,871
	**Per 100,000 Beneficiaries**	**162**	**161**	**167**	**190**	**215**

S 3: Medicare nuclear cardiac imaging activity by CPT code.

CPT Code	Description	2011	2012	2013	2014	2015
78451	Ht muscle image spect sing	62,012	52,519	44,971	41,426	40,383
78452	Ht muscle image spect mult	2,462,915	2,316,398	2,160,039	2,046,624	1,979,900
78453	Ht muscle image planar sing	2,366	2,045	1,638	1,918	1,618
78454	Ht musc image planar mult	16,628	15,149	12,770	12,568	1,184
78472	Gated heart planar single	44,392	38,275	34,705	31,878	29,198
78473	Gated heart multiple	2,875	2,501	2,070	1,852	1,777
78481	Heart first pass single	1,990	1,465	1,375	1,275	1,228
78483	Heart first pass multiple	357	273	224	299	297
78459	Heart image PET, single, metabolic	1,922	1,800	1,920	1,821	1,980
78491	Heart image PET single, perfusion	987	765	835	923	990
78492	Heart image PET multiple, perfusion	63,668	62,330	90,362	100,093	106,327
	SUM	2,660,112	2,493,520	2,350,909	2,240,677	2,164,882
	**Per 100,000 Beneficiaries**	**8,230**	**7,632**	**7,148**	**6,803**	**6,563**

S 4: Medicare echocardiography activity by CPT code.

CPT Code	Description	2011	2012	2013	2014	2015
93306	Tte w/doppler complete	7,202,932	7,102,981	7,001,658	6,980,503	7,025,217
93307	Tte w/o doppler complete	75,512	63,058	53,988	43,774	36,680
93308	Tte f-up or lmtd	170,144	181,350	197,334	220,177	252,380
93312	Echo transesophageal	248,111	256,724	272,625	276,219	282,341
93314	Echo transesophageal interpretation & report	21,794	23,158	25,658	25,152	21,156
93355	Echo transesophageal structural guidance					10,392
	SUM	7,718,493	7,627,271	7,551,263	7,545,825	7,617,774
	**Per 100,000 Beneficiaries**	**23,880**	**23,345**	**22,958**	**22,908**	**23,093**

S5: Number of Medicare (excluding Medicare Advantage Plan) beneficiaries.

	Total	Aged Enrollees	Disabled Enrollees
2011	32,322,000	26,592,000	5,730,000
2012	32,672,000	26,900,000	5,772,000
2013	32,891,000	27,108,000	5,783,000
2014	32,939,000	27,221,000	5,718,000
2015	32,987,000	27,428,000	5,559,000

Population data summed from Boards of the Trustees of the Federal Hospital Insurance and Federal Supplementary Medical Insurance Trust Funds Reports, Table IV.B2.
https://www.cms.gov/Research-Statistics-Data-and-Systems/Statistics-Trends-and-Reports/ReportsTrustFunds/TrusteesReports.html?DLSort=0&DLEntries=10&DLPage=1&DLSortDir=descending
NOTES: Persons entitled because of End Stage Renal Disease are excluded in the disabled enrollee count.
Utilization data retrieved from Physician/Supplier Procedure Master Files, curated by the AMA for use at the RUC, aka, the RUC Database.
http://www.cms.gov/Research-Statistics-Data-and-Systems/Files-for-Order/NonIdentifiableDataFiles/PhysicianSupplierProcedureSummaryMasterFile.html

S6: 72 SNOMED-CT codes selected from entire 2458 codes captured in the Diagnostic Imaging Dataset to determine non-invasive imaging activity in England.

CMR
Magnetic resonance imaging angiography of coronary arteries (procedure) (419997008)
Magnetic resonance imaging for cardiac morphology and function (procedure) (449876000)
Magnetic resonance imaging for cardiac morphology and function under stress (procedure) (449882002)
Magnetic resonance imaging for cardiac morphology and function under stress with contrast (procedure) (449883007)
Magnetic resonance imaging for cardiac morphology and function with contrast (procedure) (449879007)
Magnetic resonance imaging for cardiac morphology, function, and velocity (procedure) (449877009)
Magnetic resonance imaging for cardiac morphology, function, and velocity under stress (procedure) (449878004)
Magnetic resonance imaging for cardiac morphology, function, and velocity under stress with contrast (procedure) (449881009)
Magnetic resonance imaging for cardiac morphology, function, and velocity with contrast (procedure) (449880005)
Magnetic resonance imaging of aorta (procedure) (306992007)
Magnetic resonance imaging of aorta with contrast (procedure) (450527004)
Magnetic resonance imaging of cardiac valvular function (procedure) (432845009)
Magnetic resonance imaging of cardiac ventricular volume (procedure) (432846005)
Magnetic resonance imaging of thoracic aorta (procedure) (241666000)
Magnetic resonance imaging of thoracic aorta with contrast (procedure) (450528009)
Magnetic resonance imaging T2 mapping (procedure) (450558003)

CCT
Computed tomography angiography for transcatheter aortic valve implantation planning (procedure) (1051431000000100)
Computed tomography angiography of coronary arteries (procedure) (419545005)
Computed tomography angiography of coronary artery bypass graft (procedure) (450506009)
Computed tomography of heart (procedure) (241547009)
Computed tomography of thoracic aorta (procedure) (241548004)
Computed tomography of thoracic aorta with contrast (procedure) (444970007)
Gated computed tomography for cardiac function with contrast (procedure) (711371001)
Gated computed tomography for complex congenital heart disease with contrast (procedure) (772821000000105)
Gated computed tomography of heart with contrast (procedure) (448431001)

Nuclear
Cardiac Tc-99m methoxyisobutyl isonitrile study (procedure) (252434009)
Imaging of heart using iodine 123 meta-iodobenzylguanidine (procedure) (433218007)
Myocardial perfusion stress imaging using Thallium 201 (procedure) (431511008)
Radionuclide cardiac first pass angiography (procedure) (431942008)
Radionuclide cardiac first pass ventriculography (procedure) (448049005)
Radionuclide cardiac shunt study (procedure) (252431001)
Radionuclide cardiac study for detection of right to left lung shunt (procedure) (443340003)
Radionuclide electrocardiography gated myocardial perfusion rest and redistribution study using thallium 201 (procedure) (826711000000105)

Radionuclide electrocardiography gated myocardial perfusion rest study using technetium Tc^99m^ methoxyisobutylisonitrile (procedure) (446182004)
Radionuclide electrocardiography gated myocardial perfusion rest study using technetium Tc^99m^ tetrofosmin (procedure) (447525000)
Radionuclide electrocardiography gated myocardial perfusion rest study using thallium 201 (procedure) (431644007)
Radionuclide electrocardiography gated myocardial perfusion stress and redistribution study using thallium 201 (procedure) (840921000000100)
Radionuclide electrocardiography gated myocardial perfusion stress study using technetium Tc^99m^ methoxyisobutylisonitrile (procedure) (447586001)
Radionuclide electrocardiography gated myocardial perfusion stress study using technetium Tc^99m^ tetrofosmin (procedure) (446875000)
Radionuclide imaging of perfusion of myocardium under exercise stress (procedure) (440519002)
Radionuclide imaging of perfusion of myocardium under stress and reinjection using Thallium 201 (procedure) (440335005)
Radionuclide myocardial perfusion rest and redistribution study using thallium 201 (procedure) (826691000000108)
Radionuclide myocardial perfusion stress study using technetium Tc^99m^ methoxyisobutylisonitrile (procedure) (446876004)
Radionuclide myocardial perfusion stress study using technetium Tc^99m^ tetrofosmin (procedure) (447526004)
Radionuclide myocardial perfusion study (procedure) (252432008)
Radionuclide myocardial perfusion study using technetium Tc^99m^ tetrofosmin (procedure) (446874001)
Radionuclide ventriculography at cardiac rest (procedure) (432115009)
Radionuclide ventriculography at cardiac stress (procedure) (432155006)
Positron emission tomography electrocardiography gated myocardial rest study using fluorodeoxyglucose (procedure) (434267000)
Positron emission tomography myocardial rest imaging using fluorodeoxyglucose (procedure) (432026001)
Positron emission tomography myocardial rest study using N13 ammonia (procedure) (241441008)
Positron emission tomography myocardial stress imaging using rubidium 81 (procedure) (431901005)
Positron emission tomography myocardial stress imaging using rubidium 82 (procedure) (818291000000100)
Positron emission tomography of heart for cardiac viability using fluorodeoxyglucose (procedure) (433227008)
Positron emission tomography using nitrogen 13 ammonia with computed tomography electrocardiography gated myocardial rest study (procedure) (443277009)
Positron emission tomography using rubidium 81 with computed tomography myocardial rest imaging (procedure) (443628005)
Positron emission tomography using rubidium 81 with computed tomography myocardial stress imaging (procedure) (443629002)
Positron emission tomography with computed tomography of heart for cardiac viability using fluorodeoxyglucose (procedure) (443249000)
Single photon emission computerised tomography cardiac study using iodine 123 meta-iodobenzylguanidine (procedure) (359151000000102)

Echocardiography
Doppler ultrasonography of heart tissue (procedure) (439858009)
Exercise stress echocardiography (procedure) (433233004)
Exercise stress ultrasonography of heart with contrast (procedure) (433862009)
Focused transthoracic echocardiography in intensive care (procedure) (719508009)
Three dimensional transesophageal ultrasonography of heart (procedure) (445864005)
Transesophageal echocardiography (procedure) (105376000)
Transesophageal echocardiography with contrast (procedure) (440467009)
Transoesophageal echocardiography for complex congenital heart disease (procedure) (772551000000103)
Transthoracic echocardiography (procedure) (433236007)
Transthoracic echocardiography for complex congenital heart disease (procedure) (772531000000105)
Transthoracic three dimensional ultrasonography of heart (procedure) (434158009)
Transthoracic ultrasonography of heart with contrast (procedure) (434167009)
Ultrasonography of cardiac ventricle for resynchronization (procedure) (433033007)

S7: Recommendations containing class, level, score from current AHA/ACC, ESC clinical practice guidelines, Appropriateness Use Criteria and the Choosing Wisely programme.

	Substitutable by other (imaging) test	Guideline (ACC/AHA)	Year last updated	Class	Level	Guideline (ESC)	Year last updated	Class	Level	Appropriateness Use Criteria [2006]	Choosing Wisely [2014]
**Suspected / stable coronary artery disease**											
Pharmacological stress with CMR can be useful for patients with an intermediate to high pretest probability of obstructive ischemic heart disease, who have an uninterpretable ECG and at least moderate physical functioning or no disabling comorbidity.	Y	Stable Coronary Artery Disease	2014	IIa	B					A(7)--if intermediate pretest probability U(5)--if high pretest probability	
Pharmacological stress CMR is reasonable for patients with an intermediate to high pretest probability of ischemic heart disease, who are incapable of at least moderate physical functioning or have disabling comorbidity.	Y	Stable Coronary Artery Disease	2014	IIa	B					A(7)--if intermediate pretest probability U(5)--if high pretest probability	
Echocardiography, radionuclide imaging, CMR, and cardiac CT are not recommended for routine assessment of LV function in patients with a normal ECG, no history of myocardial infarction, no symptoms or signs suggestive of heart failure, and no complex ventricular arrhythmias.	(Y)	Stable Coronary Artery Disease	2014	III	C						
Routine reassessment (<1 year) of LV function with technologies such as echocardiography, radionuclide imaging, CMR, or cardiac CT is not recommended in patients with no change in clinical status and for whom no change in therapy is contemplated.	(Y)	Stable Coronary Artery Disease	2014	III	C						
CMR with pharmacological stress is reasonable for risk assessment in patients with stable ischemic heart disease who are able to exercise to an adequate workload but have an uninterpretable ECG.	Y	Stable Coronary Artery Disease	2014	IIa	B	Stable Coronary Artery Disease	2013	I	B		

	Substitutable by other (imaging) test	Guideline (ACC/AHA)	Year last updated	Class	Level	Guideline (ESC)	Year last updated	Class	Level	Appropriateness Use Criteria [2006]	Choosing Wisely [2014]
Pharmacological stress imaging (nuclear MPI, echocardiography, or CMR) or CCTA is not recommended for risk assessment in patients with stable ischemic heart disease who are able to exercise to an adequate workload and have an interpretable ECG.	(Y)	Stable Coronary Artery Disease	2014	III	C					I(2)--if low pretest probability. U(4)--if intermediate	
Pharmacological stress CMR is reasonable for risk assessment in patients with stable ischemic heart disease who are unable to exercise to an adequate workload regardless of interpretability of ECG.	Y	Stable Coronary Artery Disease	2014	IIa	B						
In patients with suspected stable coronary artery disease and intermediate pretest probability of 15% - 65% and LVEF =50 %, stress imaging is preferred as the initial test option if local expertise and availability permit.	Y					Stable Coronary Artery Disease	2013	I	B		
An imaging stress test is recommended as the initial test for diagnosing stable coronary artery disease if the pretest probability is between 66-85 % or if LVEF is <50 % in patients without typical angina.	Y					Stable Coronary Artery Disease		I	B		
An imaging stress test is recommended in patients with resting ECG abnormalities, which prevent accurate interpretation of ECG changes during stress.	Y					Stable Coronary Artery Disease		I	B		
Stress imaging for risk stratification is recommended in patients with a non-conclusive exercise ECG.	Y					Stable Coronary Artery Disease		I	B		
Risk stratification is recommended based on clinical assessment and the results of the stress test initially employed for making a diagnosis of stable coronary artery disease.	Y					Stable Coronary Artery Disease	2013	I	B		

	Substitutable by other (imaging) test	Guideline (ACC/AHA)	Year last updated	Class	Level	Guideline (ESC)	Year last updated	Class	Level	Appropriateness Use Criteria [2006]	Choosing Wisely [2014]
In asymptomatic adults with diabetes or asymptomatic adults with a strong family history of coronary artery disease or when previous risk assessment testing suggests high risk of coronary artery disease, such as a coronary artery calcium score of 400 or greater stress imaging tests (MPI, stress echocardiography, perfusion CMR) may be considered for advanced cardiovascular risk assessment.	Y					Stable Coronary Artery Disease		IIb	C		
In patients with stable coronary disease after a significant change in symptom level, risk stratification using stress ECG (unless they cannot exercise or display ECG changes which make the ECG non evaluable) or preferably stress imaging if local expertise and availability permit is recommended.	Y					Stable Coronary Artery Disease		I	B		
In patients with known stable coronary artery disease and a deterioration in symptoms, stress imaging is recommended for risk stratification if the site and extent of ischemia would influence clinical decision making.	Y					Stable Coronary Artery Disease		I	B		
An exercise ECG or stress imaging if appropriate is recommended in the presence of recurrent or new symptoms once instability has been ruled out.	Y					Stable Coronary Artery Disease		I	C		
Reassessment of the prognosis using stress testing may be considered in asymptomatic patients after the expiration of the period for which the previous test was felt to be valid ("warranty period").	Y					Stable Coronary Artery Disease		IIb	C		
Evaluation of Chest Pain Syndrome (Use of MR Coronary Angiography) Intermediate pre-test probability of CAD ECG interpretable AND able to exercise.	(Y)									I(2)	

	Substitutable by other (imaging) test	Guideline (ACC/AHA)	Year last updated	Class	Level	Guideline (ESC)	Year last updated	Class	Level	Appropriateness Use Criteria [2006]	Choosing Wisely [2014]
Evaluation of Chest Pain Syndrome (Use of MR Coronary Angiography) Intermediate pre-test probability of CAD -ECG uninterpretable OR unable to exercise.	(Y)									I(2)	
Evaluation of Chest Pain Syndrome (Use of MR Coronary Angiography) High pre-test probability of CAD.	(Y)									I(2)	
Don't perform stress cardiovascular magnetic resonance (CMR) in the initial evaluation of chest pain patients with low pretest probability of coronary artery disease.	(Y)										1
Risk assessment by CMR of patients with normal prior stress test (exercise, nuclear, echo, MRI)<br>● High CHD risk (Framingham)<br>● Within 1 year of prior stress test.	(Y)									I(2)	
Risk assessment by CMR of patients with equivocal prior stress test (exercise, stress SPECT, or stress echo)<br>● Intermediate CHD risk (Framingham).	N									U (6)	
Coronary angiography (catheterization or CT)<br>● Stenosis of unclear significance.	Y									A (7)	
**Acute coronary syndrome**											
Imaging with ventriculography, echocardiography, or magnetic resonance imaging should be performed to confirm or exclude the diagnosis of stress (Takotsubo) cardiomyopathy.	Y	NSTEMI	2014	I	B						

	Substitutable by other (imaging) test	Guideline (ACC/AHA)	Year last updated	Class	Level	Guideline (ESC)	Year last updated	Class	Level	Appropriateness Use Criteria [2006]	Choosing Wisely [2014]
In patients with no recurrence of chest pain, normal ECG findings and normal levels of cardiac troponin (preferably high-sensitivity), but suspected acute coronary syndrome, a non-invasive stress test (preferably with imaging) for inducible ischaemia is recommended before deciding on an invasive strategy.	Y					ACS without ST elevation	2015	I	A	U(6)	
When echocardiography is suboptimal/inconclusive, an alternative imaging method (CMR preferably) should be considered to assess LV function in patients with STEMI, both before and after discharge.	Y					STEMI	2017	IIa	C	U(6)	
Stress echo, CMR, SPECT, or PET may be used to assess myocardial ischaemia and viability, including in multivessel CAD.	Y					STEMI	2017	IIb	C		
Acute Chest Pain (Use of Vasodilator Perfusion CMR or Dobutamine Stress Function CMR). High pre-test probability of CAD ECG—ST-segment elevation and/or positive cardiac enzymes.	(N)									I(1)	3
Evaluation of Myocardial Scar (Use of Late Gadolinium Enhancement) To determine the location and extent of myocardial necrosis including 'no reflow' regions * Post-acute myocardial infarction.	N									A(7)	
**Before coronary revascularization**											
Non-invasive stress imaging (CMR, stress echocardiography, SPECT, or PET) may be considered for the assessment of myocardial ischaemia and viability in patients with HF and CAD (considered suitable for coronary revascularisation) before the decision on revascularisation.	Y					Myocardial Revascularisation	2018	IIb	B		

	Substitutable by other (imaging) test	Guideline (ACC/AHA)	Year last updated	Class	Level	Guideline (ESC)	Year last updated	Class	Level	Appropriateness Use Criteria [2006]	Choosing Wisely [2014]
Either exercise or pharmacological stress with imaging (nuclear MPI, echocardiography, or CMR) is recommended for risk assessment in patients with stable ischemic heart disease, who are being considered for revascularization of known coronary stenosis of unclear physiological significance.	Y	Stable Coronary Artery Disease	2014	I	B						
An imaging stress test should be considered to assess the functional severity of intermediate lesions on coronary arteriography.	Y					Stable Coronary Artery Disease	2013	IIa	B		
Evaluation of Myocardial Scar (Use of Late Gadolinium Enhancement) To determine viability prior to revascularization. Establish likelihood of recovery of function with revascularization (PCI or CABG) or medical therapy.	Y									A(9)	
Evaluation of Myocardial Scar (Use of Late Gadolinium Enhancement) To determine viability prior to revascularization and Viability assessment by SPECT or dobutamine echo has provided “equivocal or indeterminate” results.	N									A(9)	
**After coronary revascularization**											
Late (6 months) stress imaging test after revascularization may be considered to detect patients with restenosis after stenting or graft occlusion irrespective of symptoms.	Y					Stable Coronary Artery Disease	2013	IIb	C		
In symptomatic patients with revascularized stable coronary artery disease, stress imaging (stress echocardiography, CMR or MPS) is indicated rather than stress ECG.	Y					Stable Coronary Artery Disease	2013	I	C		
In symptomatic patients with prior revascularization (PCI or CABG), an imaging stress test should be considered.	Y					Stable Coronary Artery Disease	2013	IIa	B		

	Substitutable by other (imaging) test	Guideline (ACC/AHA)	Year last updated	Class	Level	Guideline (ESC)	Year last updated	Class	Level	Appropriateness Use Criteria [2006]	Choosing Wisely [2014]
Detection of CAD: Post-Revascularization (PCI or CABG) Evaluation of Chest Pain Syndrome (Use of MR Coronary Angiography) Evaluation of bypass grafts.	(N)									I(2)	
Detection of CAD: Post-Revascularization (PCI or CABG) Evaluation of Chest Pain Syndrome (Use of MR Coronary Angiography) History of percutaneous revascularization with stents.	(N)									I(1)	4
Surveillance by non-invasive imaging-based stress testing may be considered in high-risk patient subsets 6 months after revascularisation.	Y					Myocardial Revascularisation	2018	IIb	C		
An imaging stress test should be considered in patients with prior revascularisation over stress ECG.	Y					Myocardial Revascularisation	2018	IIa	B		
Routine non-invasive imaging-based stress testing may be considered 1 year after PCI and more than 5 years after CABG.	Y					Myocardial Revascularisation	2018	IIb	C		
**Risk stratification before non-cardiac surgery**											
Imaging stress test is recommended before high risk surgery in patients with more than two clinical risk factors and poor functional capacity (<4 METs).	Y					Non-cardiac Surgery	2014	I	C	U(6)	
Imaging stress testing may be considered before high- or intermediate-risk surgery in patients with one or two clinical risk factors and poor functional capacity (<4 METs).	Y					Non-cardiac Surgery	2014	IIb	C	U(6)	
Imaging stress testing is not recommended before low-risk surgery, regardless of the patient's clinical risk.	(Y)					Non-cardiac Surgery	2014	III	C	I (2)	2

	Substitutable by other (imaging) test	Guideline (ACC/AHA)	Year last updated	Class	Level	Guideline (ESC)	Year last updated	Class	Level	Appropriateness Use Criteria [2006]	Choosing Wisely [2014]
**Heart failure**											
CMR is recommended for the assessment of myocardial structure and function (including right heart) in subjects with poor acoustic windows and patients with complex congenital heart diseases.	N	Heart Failure	2017	IIa	C	Heart Failure	2016	I	C		
CMR with LGE should be considered in patients with dilated cardiomyopathy in order to distinguish between ischaemic and non-ischaemic myocardial damage in case of equivocal clinical and other imaging data.	N					Heart Failure	2016	IIa	C		
CMR is recommended for the characterisation of myocardial tissue in case of suspected myocarditis, amyloidosis, sarcoidosis, Chagas disease, Fabry disease, non-compaction cardiomyopathy and haemochromatosis.	N	Heart Failure	2017	IIa	B					A(8)	
Non-invasive stress imaging (CMR, stress echocardiography, SPECT, PET) may be considered for the assessment of myocardial ischaemia and viability in patients with HF and CAD (considered suitable for coronary revascularisation) before the decision on revascularisation.	N					Heart Failure	2016	IIb	B		
**Ventricular arrhythmia**											
In patients with suspected non-ischaemic cardiomyopathy from myocardial infiltrative processes, cardiac MRI with late gadolinium enhancement is useful for diagnosis.	N	Ventricular Arrhythmias	2017	I	B-NR						
In patients with suspected non-ischaemic cardiomyopathy, cardiac MRI with late gadolinium enhancement can be useful for assessing risk of SCA/SCD.	N	Ventricular Arrhythmias	2017	IIa	B-NR						

	Substitutable by other (imaging) test	Guideline (ACC/AHA)	Year last updated	Class	Level	Guideline (ESC)	Year last updated	Class	Level	Appropriateness Use Criteria [2006]	Choosing Wisely [2014]
In patients with suspected arrhythmogenic right ventricular cardiomyopathy and VA or electrocardiographic abnormalities, cardiac MRI is useful or establishing a diagnosis and risk stratification.	N	Ventricular Arrhythmias	2017	I	B-NR					A(9)	
MRI, cardiac computed tomography (CT), or radionuclide angiography can be useful in patients with ventricular arrhythmias when echocardiography does not provide accurate assessment of LV and RV function and/or evaluation of structural changes.	Y	Ventricular Arrhythmias	2017	IIa	C-EO	Ventricular Dysrhythmias	2015	IIa	B		
Pharmacological stress testing plus imaging modality is recommended to detect silent ischaemia in patients with ventricular arrhythmias who have an intermediate probability of having coronary artery disease by age or symptoms and are physically unable to perform a symptom-limited exercise test.	Y					Ventricular Dysrhythmias	2015	I	B		
**Inflammatory heart disease**											
Demonstration of persistent myocardial inflammatory infiltrates by immunohistological evidence and/or abnormal localized fibrosis by CMR after acute myocarditis may be considered as an additional indicator of increased risk of SCD in inflammatory heart disease.	N					Ventricular Dysrhythmias	2015	IIb	C		
CMR is recommended for the confirmation of myocardial involvement in pericarditis.	N					Pericardial Disease	2015	I	C		
Evaluation of myocarditis or myocardial infarction with normal coronary arteries. Positive cardiac enzymes without obstructive atherosclerosis on angiography.	N									A(8)	

	Substitutable by other (imaging) test	Guideline (ACC/AHA)	Year last updated	Class	Level	Guideline (ESC)	Year last updated	Class	Level	Appropriateness Use Criteria [2006]	Choosing Wisely [2014]
**Hypertrophic cardiomyopathy**											
CMR imaging is indicated in patients with suspected HCM when echocardiography is inconclusive for diagnosis.	N	Hypertrophic Cardiomyopathy	2011	I	B	Hypertrophic Cardiomyopathy	2014	I	C		
CMR imaging is indicated in patients with known HCM when additional information that may have an impact on management or decision making regarding invasive management, such as magnitude and distribution of hypertrophy or anatomy of the mitral valve apparatus or papillary muscles, is not adequately defined with echocardiography.	N	Hypertrophic Cardiomyopathy	2011	I	B	Hypertrophic Cardiomyopathy	2014	IIb	C		
CMR imaging is reasonable in patients with HCM to define apical hypertrophy and/or aneurysm if echocardiography is inconclusive.	N	Hypertrophic Cardiomyopathy	2011	IIa	B	Hypertrophic Cardiomyopathy	2014	IIa	C		
In selected patients with known HCM, when SCD risk stratification is inconclusive after documentation of the conventional risk factors, CMR imaging with assessment of late gadolinium enhancement (LGE) may be considered in resolving clinical decision making.	N	Hypertrophic Cardiomyopathy	2011	IIb	C						
The usefulness of the following potential SCD risk modifiers is unclear but might be considered in selected patients with HCM for whom risk remains borderline after documentation of conventional risk factors: CMR imaging with LGE.	N	Hypertrophic Cardiomyopathy	2011	IIb	C						
It is recommended that CMR studies in suspected HCM be performed and interpreted by teams experienced in cardiac imaging and in the evaluation of heart muscle disease.	N					Hypertrophic Cardiomyopathy	2014	I	B		

	Substitutable by other (imaging) test	Guideline (ACC/AHA)	Year last updated	Class	Level	Guideline (ESC)	Year last updated	Class	Level	Appropriateness Use Criteria [2006]	Choosing Wisely [2014]
CMR may be considered every 5 years in clinically stable patients, or every 2–3 years in patients with progressive disease.	N					Hypertrophic Cardiomyopathy	2014	IIb	C		
**Athlete's heart**											
Extended monitoring (including MRI) can be beneficial for athletes with unexplained exertional syncope after an initial cardiovascular evaluation.	Y	Syncope	2017	IIa	C-LD						
For prevention of sudden cardiac death in athletes, upon identification of ECG abnormalities suggestive of structural heart disease, echocardiography and/or CMR imaging is recommended.	Y					Ventricular Dysrhythmias	2015	I	C		
**Storage disease**											
CMR imaging may be considered in patients with LV hypertrophy and the suspicion of alternative diagnoses to HCM, including cardiac amyloidosis, Fabry disease and genetic phenocopies such as LAMP2 cardiomyopathy.	N	Hypertrophic Cardiomyopathy	2011	IIb	C	Hypertrophic Cardiomyopathy	2014	IIa	C		
**Pericardial diseases**											
CMR is second-level testing for diagnostic workup in pericarditis.	N					Pericardial Disease	2011	I	C	A(8)	
CMR should be considered in suspected cases of loculated pericardial effusion, pericardial thickening and masses, as well as associated chest abnormalities.	N					Pericardial Disease	2011	IIa	C		
CMR is indicated as second-level imaging technique to assess pericardial thickness, degree and extension of pericardial involvement for the diagnosis of constrictive pericarditis.	N					Pericardial Disease	2011	IIa	C		

	Substitutable by other (imaging) test	Guideline (ACC/AHA)	Year last updated	Class	Level	Guideline (ESC)	Year last updated	Class	Level	Appropriateness Use Criteria [2006]	Choosing Wisely [2014]
Empiric anti-inflammatory therapy may be considered in cases with transient or new diagnosis of constrictive pericarditis with concomitant evidence of pericardial inflammation (i.e. pericardial enhancement on CMR).	N					Pericardial Disease	2011	IIb	C		
**Pregnancy**											
CMR (without gadolinium) should be considered if echocardiography is insufficient for diagnosis.	N					Cardiovascular Disease in Pregnancy	2011	IIa	C		
Imaging of the entire aorta (CT/CMR) should be performed before pregnancy in patients with Marfan syndrome or other known aortic disease.	Y					Cardiovascular Disease in Pregnancy	2011	I	C		
**Peripheral Vascular Disease**											
Duplex ultrasound, computed tomography angiography (CTA), or magnetic resonance angiography (MRA) of the lower extremities is useful to diagnose anatomic location and severity of stenosis for patients with symptomatic peripheral artery disease in whom revascularization is considered.	Y	Peripheral Artery Disease	2016	I	B-NR	Peripheral Artery Disease	2011	I	A		
Invasive and noninvasive angiography (ie, CTA, MRA) should not be performed for the anatomic assessment of patients with asymptomatic peripheral artery disease.	(Y)	Peripheral Artery Disease	2016	III	B-R						
In patients with acute, focal ischemic neurological symptoms corresponding to the territory supplied by the left or right internal carotid artery, magnetic resonance angiography (MRA) or computed tomography angiography (CTA) is indicated to detect carotid stenosis when sonography either cannot be obtained or yields equivocal or otherwise nondiagnostic results.	Y	Carotid and Vertebral Artery	2011	I	C						

	Substitutable by other (imaging) test	Guideline (ACC/AHA)	Year last updated	Class	Level	Guideline (ESC)	Year last updated	Class	Level	Appropriateness Use Criteria [2006]	Choosing Wisely [2014]
When an extracranial source of ischemia is not identified in patients with transient retinal or hemispheric neurological symptoms of suspected ischemic origin, CTA, MRA, or selective cerebral angiography can be useful to search for intracranial vascular disease.	Y	Carotid and Vertebral Artery	2011	IIa	C						
When the results of initial noninvasive imaging are inconclusive, additional examination by use of another imaging method is reasonable. In candidates for revascularization, MRA or CTA can be useful when results of carotid duplex ultrasonography are equivocal or indeterminate.	Y	Carotid and Vertebral Artery	2011	IIa	C						
MRA without contrast is reasonable to assess the extent of disease in patients with symptomatic carotid atherosclerosis and renal insufficiency or extensive vascular calcification.	N	Carotid and Vertebral Artery	2011	IIa	C						
When complete carotid arterial occlusion is suggested by duplex ultrasonography, MRA, or CTA in patients with retinal or hemispheric neurological symptoms of suspected ischemic origin, catheter-based contrast angiography may be considered to determine whether the arterial lumen is sufficiently patent to permit carotid revascularization.	Y	Carotid and Vertebral Artery	2011	IIb	C						
Noninvasive imaging by CTA or MRA for detection of vertebral artery disease should be part of the initial evaluation of patients with neurological symptoms referable to the posterior circulation and those with subclavian steal syndrome.	Y	Carotid and Vertebral Artery	2011	I	C						

	Substitutable by other (imaging) test	Guideline (ACC/AHA)	Year last updated	Class	Level	Guideline (ESC)	Year last updated	Class	Level	Appropriateness Use Criteria [2006]	Choosing Wisely [2014]
In patients whose symptoms suggest posterior cerebral or cerebellar ischemia, MRA or CTA is recommended rather than ultrasound imaging for evaluation of the vertebral arteries.	Y	Carotid and Vertebral Artery	2011	I	C						
Contrast-enhanced CTA, MRA, and catheter-based contrast angiography are useful for diagnosis of cervical artery dissection.	Y	Carotid and Vertebral Artery	2011	I	C						
Duplex ultrasound (DUS, as first-line imaging), CTA and/or MRA are recommended for evaluating the extent and severity of extracranial carotid stenoses.	Y					Peripheral Artery Disease	2017	I	B		
When carotid artery stenosis is being considered, it is recommended that any DUS study be followed by either MRA or CTA to evaluate the aortic arch as well as the extra- and intracranial circulation.	Y					Peripheral Artery Disease	2017	I	B		
When carotid endarterectomy (CEA) is considered, it is recommended that the DUS stenosis estimation be corroborated by either MRA or CTA (or by a repeat DUS study performed in an expert vascular laboratory).	Y	Carotid and Vertebral Artery	2011	IIa	C	Peripheral Artery Disease	2017	I	B		
Duplex ultrasounds (DUS, as first-line), CTA and MRA are recommended imaging modalities to establish a diagnosis of renal artery disease.	Y					Peripheral Artery Disease	2017	I	B		
Duplex ultrasound is indicated as a first-line imaging method to confirm lower extremity arterial disease lesions.	Y					Peripheral Artery Disease	2017	I	C		
DUS and/or CTA and/or MRA are indicated for anatomical characterisation of lower extremity arterial disease lesions and guidance of optimal revascularisation strategy.	Y					Peripheral Artery Disease	2017	I	C		

	Substitutable by other (imaging) test	Guideline (ACC/AHA)	Year last updated	Class	Level	Guideline (ESC)	Year last updated	Class	Level	Appropriateness Use Criteria [2006]	Choosing Wisely [2014]
**Aortovascular disease**											
Aortic magnetic resonance angiography or CT angiography is indicated in patients with a bicuspid aortic valve when morphology of the aortic sinuses, sinotubular junction, or ascending aorta cannot be assessed accurately or fully by echocardiography.	Y	Valvular Disease	2017	I	C	Aortic Disease	2014	I	C		
Serial evaluation of the size and morphology of the aortic sinuses and ascending aorta by echocardiography, CMR, or CT angiography is recommended in patients with a bicuspid aortic valve and an aortic diameter greater than 4.0 cm, with the examination interval determined by the degree and rate of progression of aortic dilation and by family history. In patients with an aortic diameter greater than 4.5 cm, this evaluation should be performed annually.	Y	Valvular Disease	2017	I	C						
Urgent and definitive imaging of the aorta using transesophageal echocardiogram, computed tomographic imaging, or magnetic resonance imaging is recommended to identify or exclude thoracic aortic dissection in patients at high risk for the disease by initial screening.	Y	Thoracic Aorta	2010	I	B						
The initial evaluation of Takayasu arteritis or giant cell arteritis should include thoracic aorta and branch vessel computed tomographic imaging or magnetic resonance imaging to inves- tigate the possibility of aneurysm or occlusive disease in these vessels.	Y	Thoracic Aorta	2010	I	C						

	Substitutable by other (imaging) test	Guideline (ACC/AHA)	Year last updated	Class	Level	Guideline (ESC)	Year last updated	Class	Level	Appropriateness Use Criteria [2006]	Choosing Wisely [2014]
For patients with isolated aortic arch aneurysms less than 4.0 cm in diameter, it is reasonable to reimage using computed tomographic imaging or magnetic resonance imaging, at 12-month intervals, to detect enlargement of the aneurysm.	Y	Thoracic Aorta	2010	IIa	C						
For patients with isolated aortic arch aneurysms 4.0 cm or greater in diameter, it is reasonable to reimage using computed tomographic imaging or magnetic resonance imaging, at 6-month intervals, to detect en largement of the aneurysm.	Y	Thoracic Aorta	2010	IIa	C						
For imaging of pregnant women with aortic arch, descending, or abdominal aortic dilatation, magnetic resonance imaging (without gadolinium) is recommended over computed tomographic imaging to avoid exposing both the mother and fetus to ionizing radiation. Transesophageal echocardiogram is an option for imaging of the thoracic aorta.	N	Thoracic Aorta	2010	I	C	CV Disease in Pregnancy	2011	I	C		
Computed tomographic imaging or magnetic resonance imaging of the thoracic aorta is reasonable after a Type A or B aortic dissection or after prophylactic repair of the aortic root/ ascending aorta.	Y	Thoracic Aorta	2010	IIa	C						
Computed tomographic imaging or magnetic resonance imaging of the aorta is reasonable at 1, 3, 6, and 12 months postdissection and, if stable, annually thereafter so that any threatening enlargement can be detected in a timely fashion.	Y	Thoracic Aorta	2010	IIa	C						

	Substitutable by other (imaging) test	Guideline (ACC/AHA)	Year last updated	Class	Level	Guideline (ESC)	Year last updated	Class	Level	Appropriateness Use Criteria [2006]	Choosing Wisely [2014]
If a thoracic aortic aneurysm is only moderate in size and remains relatively stable over time, magnetic resonance imaging instead of computed tomographic imaging is reasonable to minimize the patient's radiation exposure.	N	Thoracic Aorta	2010	IIa	C						
MRI for detection of vascular plaque is not recommended for cardiovascular risk assessment in asymptomatic adults.	(N)	Risk Assessment	2010	III	C						5
In stable patients with a suspicion of acute aortic syndrome, CMR is recommended (or should be considered) according to local availability and expertise.	N					Aortic Disease	2014	I	C		
In case of initially negative imaging with persistence of suspicion of acute aortic syndrome, repetitive imaging (CT or CMR) is recommended.	Y					Aortic Disease	2014	I	C		
In case of uncomplicated Type B aortic dissection treated medically, repeated imaging (CT or CMR) during the first days is recommended.	Y					Aortic Disease	2014	I	C		
In uncomplicated Type B intramural hematoma, repetitive imaging (CMR or CT) is indicated.	Y					Aortic Disease	2014	I	C		
In uncomplicated Type B penetrating aortic ulcer, repetitive imaging (CMR or CT) is indicated.	Y					Aortic Disease	2014	I	C		
In the case of aortic diameter >50 mm or an increase >3 mm/year measured by echocardiography, confirmation of the measurement is indicated, using another imaging modality (CT or CMR).	Y					Aortic Disease	2014	I	C		
Contrast CT or CMR is recommended to confirm the diagnosis of chronic aortic dissection.	Y					Aortic Disease	2014	I	C	A(8)	
For follow-up after (T) EVAR in young patients, CMR should be preferred to CT for magnetic resonance-compatible stent grafts, to reduce radiation exposure.	N					Aortic Disease	2014	IIa	C		

	Substitutable by other (imaging) test	Guideline (ACC/AHA)	Year last updated	Class	Level	Guideline (ESC)	Year last updated	Class	Level	Appropriateness Use Criteria [2006]	Choosing Wisely [2014]
**Valvular heart disease**											
Noninvasive imaging (stress nuclear/positron emission tomography, CMR, or stress echocardiography), cardiac CT angiography, or cardiac catheterization, including coronary arteriography, is useful to establish etiology of chronic secondary MR (stages B to D) and/or to assess myocardial viability, which in turn may influence management of functional MR.	Y	Valvular Disease	2017	I	C						
CMR is indicated in patients with moderate or severe AR (stages B, C, and D) and suboptimal echocardiographic images for the assessment of LV systolic function, systolic and diastolic volumes, and measurement of AR severity.	N	Valvular Disease	2017	I	B						
CMR is indicated in patients with chronic primary MR to assess LV and RV volumes, function, or MR severity and when these issues are not satisfactorily addressed by TTE.	N	Valvular Disease	2017	I	B						
CMR or real-time 3D echocardiography may be considered for assessment of right ventricular systolic function and systolic and diastolic volumes in patients with severe tricuspid regurgitation (stages C and D) and suboptimal 2D echocardiograms.	Y	Valvular Disease	2017	IIb	C						
Characterization of native and prosthetic cardiac valves—including planimetry of stenotic disease and quantification of regurgitant disease. Patients with technically limited images from echocardiogram or TEE.	N									A(8)	

	Substitutable by other (imaging) test	Guideline (ACC/AHA)	Year last updated	Class	Level	Guideline (ESC)	Year last updated	Class	Level	Appropriateness Use Criteria [2006]	Choosing Wisely [2014]
In patients with inadequate echocardiographic quality of discrepant results, cardiac magnetic resonance (CMR) should be used to assess the severity of valvular lesions, particularly regurgitant lesions, and to assess ventricular volumes, systolic function, abnormalities of the ascending aorta and myocardial fibrosis.	N					Valvular Heart Disease	2017				
If the ascending aorta is dilated (> 40mm) it is recommended to perform CT or MRI.	Y					Valvular Heart Disease	2017				
MSCT is the preferred imaging tool to assess the anatomy and dimensions of the aortic root, size and shape of the aortic valve annulus, its distance to the coronary ostia, the distribution of calcifications and the number of aortic valve cusps… CMR - as an alternative technique - is, in this content, inferior to MSCR...	Y					Valvular Heart Disease	2017				
CMR is the preferred method for evaluating RV size and function and represents the gold standard for assessing RV volume and function.	Y					Valvular Heart Disease	2017				
**Congenital heart disease**											
Diagnostic and interventional procedures, including imaging (ie, echocardiography, MRI, or CT, advanced cardiac catheterization, and electrophysiology procedures for adults with complex and moderate CHD should be performed in a regional ACHD center with appropriate experience in CHD and in a laboratory with appropriate personnel and equipment. Personnel performing such procedures should work as part of a team with expertise in the surgical and transcatheter management of patients with CHD.	Y	ACHD	2008	I	C					A(9)	

	Substitutable by other (imaging) test	Guideline (ACC/AHA)	Year last updated	Class	Level	Guideline (ESC)	Year last updated	Class	Level	Appropriateness Use Criteria [2006]	Choosing Wisely [2014]
In patients with ACHD who have or who are at risk of developing RV enlargement and dysfunction, serial CMR is recommended for quantitative assessment of RV size and function.	N	ACHD	2018	I	B-NR						
CMR can be useful in the initial evaluation and serial assessment of selected patients with CHD based on anatomic complexity and clinical status.	N	ACHD	2018	IIa	C-LD						
CMR, CCT, and/or TEE are useful to evaluate pulmonary venous connections in adults with ASD.	Y	ACHD	2018	I	B-NR						
CMR or CTA is recommended for evaluation of partial anomalous pulmonary venous connection.	Y	ACHD	2018	I	B-NR						
Adults with bicuspid aortic valve should be evaluated for coarctation of the aorta by clinical examination and imaging studies.	Y	ACHD	2018	I	B-NR						
Aortic imaging using TTE, TEE, CMR, or CTA is recommended in adults with Williams syndrome or patients suspected of having supravalvular aortic stenosis.	Y	ACHD	2018	I	C-LD						
Initial and follow-up aortic imaging using CMR or CTA is recommended in adults with coarctation of the aorta, including those who have had surgical or catheter intervention.	Y	ACHD	2018	I	B-NR						
In adults with Ebstein anomaly, CMR can be useful to determine anatomy, RV dimensions, and systolic function.	N	ACHD	2018	IIa	B-NR						
Coronary angigraphy, using catheteristion, CT, or CMR, is recommended for evaluation of anomalous coronar artery.	Y	ACHD	2018	I	C-LD						
CMR is useful to quantify ventricular size and function, pulmonary valve function, pulmonary artery anatomy, and left heart abnormalities in patients with repaired TOF.	N	ACHD	2018	I	B-NR						

	Substitutable by other (imaging) test	Guideline (ACC/AHA)	Year last updated	Class	Level	Guideline (ESC)	Year last updated	Class	Level	Appropriateness Use Criteria [2006]	Choosing Wisely [2014]
Adults with d-TGA with atrial switch repair should undergo annual imaging with either echocardiography or CMR to evaluate for common long-term complications of the atrial switch.	Y	ACHD	2018	I	C-EO						
Baseline and serial imaging with either echocardiography or CMR should be performed in adults with d-TGA with arterial switch who have neoaortic dilation, valve dysfunction, or PA or branch PA stenosis or ventricular dysfunction.	Y	ACHD	2018	I	C-LD						
It is reasonable to perform anatomic evaluation of coronary artery patency (catheter angiography, or CT or MR angiography) in asymptomatic adults with d-TGA with arterial switch.	Y	ACHD	2018	IIa	B-NR						
CMR is reasonable in adults with CCTGA to determine systemic RV dimensions and systolic function.	N	ACHD	2018	IIa	C-LD						
Adults after Fontan palliation should be evaluated annually with either echocardiography or CMR.	Y	ACHD	2018	I	C-EO						
All patients with prior Fontan type of repair should have periodic echocardiographic and/or magnetic resonance examinations performed by staff with expertise in ACHD.	Y	ACHD	2008	I	C						
Independent of the pressure gradient, hypertensive patients with ≥50% aortic narrowing relative to the aortic diameter at the diaphragm level (on CMR, CT, or invasive angiography) should be considered for intervention.	Y					GUCHD	2010	IIa	C		
Independent of the pressure gradient and presence of hypertension, patients with ≥50% aortic narrowing relative to the aortic diameter at the diaphragm level (on CMR, CT, or invasive angiography) may be considered for intervention.	Y					GUCHD	2010	IIb	C		

	Substitutable by other (imaging) test	Guideline (ACC/AHA)	Year last updated	Class	Level	Guideline (ESC)	Year last updated	Class	Level	Appropriateness Use Criteria [2006]	Choosing Wisely [2014]
**Syncope**											
Computed tomography (CT) or magnetic resonance imaging (MRI) may be useful in selected patients presenting with syncope of suspected cardiac etiology.	Y	Syncope	2017	IIb	B-NR						
MRI and CT of the head are not recommended in the routine evaluation of patients with syncope in the absence of focal neurological findings or head injury that support further evaluation.	(Y)	Syncope	2017	III	B-NR						
**Pulmonary embolism**											
MR angiography should not be used to rule out pulmonary embolism.	(N)					Acute Pulmonary Embolism	2014	III	A		
**Others in appropriateness criteria**											
Evaluation of Ventricular and Valvular Function Procedures may include LV/RV mass and volumes, MR angiography, quantification of valvular disease, and delayed contrast enhancement Quantification of LV function Discordant information that is clinically significant from prior tests.	N									A(8)	
Evaluation of Intra- and Extra-Cardiac Structures Evaluation of cardiac mass (suspected tumor or thrombus) use of contrast for perfusion and enhancement.	N									A(9)	
Evaluation of pulmonary veins prior to radiofrequency ablation for atrial fibrillation. Left atrial and pulmonary venous anatomy including dimensions of veins for mapping purposes.	Y									A(8)	

Y=alternative imaging tests could be ordered for a given recommendation that is not class III or category Inappropriate or from Choosing Wisely. (Y) = alternative imaging tests could be ordered for a given recommendation but is not recommended anyway (class III or category Inappropriate or from Choosing Wisely). N= no alternative imaging test could be ordered for a given recommendation that is not class III or category Inappropriate or from Choosing Wisely. (N) = no alternative imaging test could be ordered for a given recommendation but is not recommended anyway (class III or category Inappropriate or from Choosing Wisely

S8: Cardiac non-invasive imaging activity per 100,000 beneficiary use for Medicare in US and NHS in England (percentage of total activity per year) – NHS activity adjustment rate of (a) 50% and (b) 70%.

(a)

Modality	USA 2011	England (2012/13)	USA 2012	England (2013/14)	USA 2013	England (2014/15)	USA 2014	England (2015/16)	USA 2015	England (2016/17)
Cardiac CT	163	142	161	180	167	212	190	250	215	317
	0.48%	2.26%	0.50%	2.69%	0.53%	2.98%	0.61%	3.27%	0.69%	3.84%
Cardiac MR	48	40	51	62	54	75	58	81	62	97
(target 227.5/year/100,000)	0.14%	0.64%	0.16%	0.92%	0.17%	1.05%	0.19%	1.06%	0.20%	1.17%
Echocardiography	25,213	5,500	24,631	5,868	24,180	6,279	24,074	6,782	24,215	7,331
	74.92%	87.40%	75.85%	87.60%	76.64%	87.98%	77.35%	88.74%	77.97%	88.71%
Nuclear	8,230	610	7,632	589	7,148	570	6,803	530	6,563	519
	24.45%	9.70%	23.50%	8.79%	22.66%	7.98%	21.86%	6.93%	21.13%	6.28%
**Total**	**33,654**	**6,293**	**32,475**	**6,699**	**31,549**	**7,137**	**31,125**	**7,642**	**31,055**	**8,263**

(b)

Modality	USA 2011	England (2012/13)	USA 2012	England (2013/14)	USA 2013	England (2014/15)	USA 2014	England (2015/16)	USA 2015	England (2016/17)
Cardiac CT	163	199	161	252	167	297	190	350	215	444
	0.48%	2.26%	0.50%	2.69%	0.53%	2.98%	0.61%	3.27%	0.69%	3.84%
Cardiac MR	48	56	51	87	54	105	58	113	62	135
(target 227.5/year/100,000)	0.14%	0.64%	0.16%	0.92%	0.17%	1.05%	0.19%	1.06%	0.20%	1.17%
Echocardiography	25,213	7,700	24,631	8,215	24,180	8,791	24,074	9,495	24,215	10,263
	74.92%	87.40%	75.85%	87.60%	76.64%	87.98%	77.35%	88.74%	77.97%	88.71%
Nuclear	8,230	854	7,632	824	7,148	798	6,803	741	6,563	726
	24.45%	9.70%	23.50%	8.79%	22.66%	7.98%	21.86%	6.93%	21.13%	6.28%
**Total**	**33,654**	**8,810**	**32,475**	**9,378**	**31,549**	**9,992**	**31,125**	**10,699**	**31,055**	**11,569**

The total (sum) of the components may contain rounding errors; Data sources as described in Figures 1-3. Activty for England restricted to beneficiaries aged 65 years and older.

S9: Cardiac non-invasive imaging activity per 100,000 beneficiary use for Medicare in US and NHS in England (percentage of total activity per year) – whole NHS population selected as denominator (54.3 million).

**Modality**	**USA 2011**	**England (2012/13)**	**USA 2012**	**England (2013/14)**	**USA 2013**	**England (2014/15)**	**USA 2014**	**England (2015/16)**	**USA 2015**	**England (2016/17)**
Cardiac CT	163<br>0.48%	53<br>2.26%	161<br>0.50%	67<br>2.69%	167<br>0.53%	79<br>2.98%	190<br>0.61%	93<br>3.27%	215<br>0.69%	118<br>3.84%
Cardiac MR (target 227.5/year/100,000)	48<br>0.14%	15<br>0.64%	51<br>0.16%	23<br>0.92%	54<br>0.17%	28<br>1.06%	58<br>0.19%	30<br>1.06%	62<br>0.20%	36<br>1.17%
Echocardiography	25,213<br>74.92%	2,046<br>87.44%	24,631<br>75.85%	2,183<br>87.64%	24,180<br>76.65%	2,336<br>88.02%	24,074<br>77.35%	2,523<br>88.74%	24,215<br>77.97%	2,727<br>88.71%
Nuclear	8,230<br>24.46%	227<br>9.70%	7,632<br>23.50%	219<br>8.79%	7,148<br>22.66%	212<br>7.99%	6,803<br>21.86%	197<br>6.93%	6,563<br>21.13%	193<br>6.28%
**Total**	**33,653**	**2,340**	**32,474**	**2,491**	**31,548**	**2,654**	**31,124**	**2,843**	**31,055**	**3,074**

The total (sum) of the components may contain rounding errors; Data sources as described in Figures 1-3.
